# Can a smartphone app improve medical trainees’ knowledge of antibiotics?

**DOI:** 10.5116/ijme.5a11.8422

**Published:** 2017-11-30

**Authors:** Michael Fralick, Reem Haj, Dhruvin Hirpara, Karen Wong, Matthew Muller, Larissa Matukas, John Bartlett, Elizabeth Leung, Linda Taggart

**Affiliations:** 1 Department of Medicine, Division of General Internal Medicine, University of Toronto, Toronto, Canada; 2 Department of Pharmacy, St Michael's Hospital, Toronto, Canada; 3 Faculty of Medicine, University of Toronto, Toronto, Canada; 4 Division of Infectious Diseases, Department of Medicine, University of Toronto, Toronto, Canada; 5 Department of Laboratory Medicine and Pathobiology, University of Toronto; 6 App Developer

**Keywords:** Antimicrobial stewardship, smartphone, app, antibiogram, Canada

## Abstract

**Objectives:**

To determine whether a smartphone app, containing local bacterial resistance patterns (antibiogram) and treatment guidelines, improved knowledge of prescribing antimicrobials among medical trainees.

**Methods:**

We conducted a prospective, controlled, pre-post study of medical trainees with access to a smartphone app (app group) containing our hospital’s antibiogram and treatment guidelines compared to those without access (control group). Participants completed a survey which included a knowledge assessment test (score range, 0 [lowest possible score] to 12 [highest possible score]) at the start of the study and four weeks later. The primary outcome was change in mean knowledge assessment test scores between week 0 and week 4. Change in knowledge assessment test scores in the app group were compared to the difference in scores in the control group using multivariable linear regression.

**Results:**

Sixty-two residents and senior medical students participated in the study. In a multivariable analysis controlling for sex and prior knowledge, app use was associated with a 1.1 point (95% CI: 0.10, 2.1) [β = 1.08, t(1) = 2.08, p = 0.04]  higher change in knowledge score compared to the change in knowledge scores in the control group. Among those in the app group, 88% found it easy to navigate, 85% found it useful, and about one- quarter used it daily.

**Conclusions:**

An antibiogram and treatment algorithm app increased knowledge of prescribing antimicrobials in the context of local antibiotic resistance patterns. These findings reinforce the notion that smartphone apps can be a useful and innovative means of delivering medical education.

## Introduction

Antimicrobial stewardship promotes the appropriate selection, dose, route, and duration of antimicrobial therapy.^1^ A recent Cochrane review demonstrated that antimicrobial stewardship programs were associated with a reduction in the duration of antimicrobial therapy, hospital length of stay, and possibly *Clostridium difficile* infection.^2^ Hospitals often develop institution-specific treatment algorithms in the context of their local antibiogram as part of an antimicrobial stewardship intervention. However, multiple studies have shown that clinicians are usually unaware these resources exist or cannot easily access this information.^3^^,^^4^

Smartphone use amongst health-care professionals has rapidly increased over the past ten years.^5^^,^^6^ Approximately 80% of doctors and 85% of medical trainees use smartphones.^7^ For this reason, they represent an innovative opportunity in the field of medical education.^7^^-^^9^ For example, a smartphone application, or 'app', that provides point-of-care information about local antibiotic resistance patterns and treatment guidelines could provide trainees accurate and up to date information from the patient's bedside. A previous study demonstrated that access to an antibiotic decision management guide on the internet improved prescribing accuracy amongst critical care fellows.^8^ One limitation of this study, and similar studies,^9^ is that these tools are seldom evaluated from a medical education standpoint. When medical apps have been evaluated, the studies have been limited by inadequate, or non-existent, control groups.^10^^-^^12^ Given these issues, we developed a smartphone app and prospectively evaluated it.

This study aimed to assess the change in knowledge of prescribing antimicrobials (e.g., antibiotics, antivirals) among medical trainees. Specifically, our research question was: Does access to a smartphone app improve medical trainees' knowledge of antimicrobials compared to medical trainees without access to the smartphone app?  We also evaluated whether the app improved confidence in prescribing, made antibiogram and treatment data more accessible, and was easy to use. We hypothesized that the smartphone app would be associated with enhanced antibiotic-related knowledge, improved confidence in prescribing and that the information contained in the app would be accessible and easy to use.

## Methods

### Study design

We conducted a prospective, controlled pre-post study between May 1, 2015, and September 24, 2015, on the general internal medicine ward at St. Michael's Hospital. St. Michael's Hospital is a tertiary care teaching hospital in Toronto, Ontario. The general internal medicine ward is a 66-bed unit cared for by five medical teams. Each team is comprised of two senior medical students (i.e., in their final 1-2 years of medical school), four resident physicians and a staff physician. An individual team will care for approximately 20 patients each day.

Senior medical students and residents rotating on general internal medicine at St. Michael's Hospital were recruited to participate in the study. Participants enrolled between May 1, 2015, and June 30, 2015, did not have access to the smartphone app (control group) while those enrolled between August 4, 2015, and September 24, 2015, had access to the app (app group). Participants were excluded if they did not have either an Apple or Android-based smartphone. Individuals could not participate in both the control group and the app group. All participants who completed the study received a $10 gift card. The study was approved by the institutional research ethics board.

### Baseline survey

Participants in the control group and app group completed the baseline survey (Appendix). This was a 27-item survey divided into four sections: demographics, knowledge assessment, self-reported confidence, and data accessibility. The demographic section included information about the participant's sex, smartphone type, training program, and level of training. Knowledge assessment was comprised of a total of 12 multiple choice or true or false questions developed for this study (Appendix). The knowledge assessment questions were trialed on a group of 30 medical students and residents at a separate teaching hospital to ensure the questions were clear, unambiguous, and had only one correct answer. Self-reported confidence was determined using a 5-point Likert scale used previously.^11^^,^^13^ The accessibility section of the survey assessed how often participants were using hospital antibiogram data and treatment algorithms at baseline. This was assessed because prior to the development of the app similar information was already available on the hospital's local intranet.

**Table 1:** Baseline characteristics of enrolled participants

Variables	Control Group (n=30)	App Group (n=32)	p-value
Sex			
Male	21 (70%)	13 (40%)	p=0.02
Level of training			
Senior medical student	13 (43%)	18 (56%)	p=0.59
Year 1 resident	8 (27%)	9 (28%)	
Year 2 resident	5 (17%)	3 (9%)	
Year 3 resident	4 (13%)	2 (6%)	
Specialty			
Internal Medicine	12 (40%)	8 (25%)	p=0.49
Family Medicine	3 (10%)	2 (6%)	
Surgery	1 (3%)	0 (0%)	
Other^*^	14 (46%)	22 (69%)	
Smartphone type			
Android	12 (40%)	13 (41%)	p=0.96
Apple	18 (60%)	19 (59%)	
Aware of hospital antibiograms			
Yes	12 (40%)	10 (32%)	p=0.47

*Included primarily medical students and a small proportion of psychiatry residents

### Follow-up survey

Participants in both groups completed the follow-up survey approximately 30 days after completing the baseline survey. The follow-up survey was identical to the baseline survey with the exception that for the app group, the follow-up survey had an additional section to assess the usability of the app. The follow-up survey was paper-based, and participants did not have access to their phone or any other resources during this time.

### Statistical analysis

The primary outcome was the change from baseline in the knowledge score (score range, 0 [lowest possible score] to 12 [highest possible score]) for the app group compared to the control group.

The secondary outcome was the change from baseline in self-reported confidence (score range, 1 [not at all confident] to 5 [very confident]). To account for confounding factors (i.e., sex, baseline knowledge, baseline confidence) both unadjusted and adjusted multivariate linear regression analyses were performed for analysis of the primary and secondary outcomes. Descriptive statistics were used to characterize participant-level characteristics and app use. Continuous data were compared with the Student's t-test with unequal variance, and categorical data were compared with the chi-square test. The analyses were conducted using SAS/STAT®  14.1 software (SAS Institute Inc., Cary, NC). All reported p-values were two-tailed.

**Table 2:** Comparison of mean knowledge scores and overall mean confidence scores between the control group and the app group

Variable	Control Group			App Group			Mean change-in-score for app group compared to control		
	Baseline (n=30)	Follow-up (n=26)	p value	Baseline (n=32)	Follow-up (n=27)	p value			
Knowledge scores (Mean, Standard Deviation)	7.1 (1.7)	7.5 (2.0)	p=0.2^a^	6.2 (2.1)	8.1 (2.2)	p<0.01^b^	Unadjusted 1.5 (95% CI: 0.46, 2.48) p=0.006		
							Adjusted^*^ 1.1 (95% CI: 0.10, 2.1) p=0.04		
Confidence in prescribing			p<0.01^a^			p<0.01^b^	Unadjusted 0.18 (p=0.34)		
							Adjusted^**^ -0.03 (p=0.86)		
Very confident (5)	0%	4%		3%	0%				
Confident (4)	13%	15.0%		9%	11%				
Neutral (3)	60%	62.0%		31%	56%				
Not very confident (2)	27%	20.0%		34%	30%				
Not at all confident (1)	0%	0.0%		22%	4%				

^a^Comparing baseline to follow-up within the control group

^b^Comparing baseline to follow-up within the app group

^*^adjusted multivariable linear regression analysis: comparing the change-in scores adjusted for baseline knowledge and sex

^**^adjusted multivariable linear regression analysis: comparing the change-in scores adjusted for baseline confidence and sex

## Results

Of the 85 medical trainees approached, 62 (73%) participated in the study. Most participants were male, approximately half were residents, and most used an Apple smartphone (Table 1). Of the residents, 17 (55%) were in first year, 8 (26%) in second year, and 6 (19%) in third year. This included 20 (65%) internal medicine residents, five family medicine residents (16%), one surgical resident 1 (3%); the remainder were from other specialties. The remaining participants were senior medical students.

At the time of study enrollment only 35% of participants were aware that St Michael's Hospital had an antibiogram. Amongst trainees in the control group, 20% reported the use of an antibiogram in the 30-days prior to enrolling in the study, and by the end of the study, 31% had used the St Michael's Hospital antibiogram. Amongst trainees in the app group, 22% reported the use of an antibiogram in the 30 days prior to enrolling in the study, and by the end of the study, 81% had used the St Michael's Hospital antibiogram.

The average number of questions answered correctly on the knowledge assessment test for the app group significantly improved over the duration of the study (6.2 points, SD=2.1 vs. 8.1 points, SD=2.2, t(26)=4.6, p=0.0001) but did not improve in the control group (7.1 points, SD=1.7 vs. 7.5 points, SD=2.0, t(25)=-1.2, p=0.23) (Table 2). In the unadjusted linear regression analysis, use of the app was associated with a 1.5 point (95% CI: 0.46, 2.48) [β = 1.46, t(1)=2.86, p=0.006] higher change in knowledge score compared to the control group. In the adjusted multivariable linear regression analysis, app use was associated with a 1.1 point (95% CI: 0.10, 2.1) [β = 1.08, t(1) = 2.08, p = 0.04] higher change in knowledge score compared to the control group (Table 2). Self-reported confidence in prescribing antibiotics improved over the duration of the study for the app group, but the change in confidence between the two groups was not statistically different in the unadjusted and adjusted analyses (Table 2).

Amongst app users, nearly 90% found it easy to use and 85% agreed that it was useful (Table 3). One-quarter of participants used the app daily or multiple times a day and 41% used it weekly.

**Table 3:** Self-reported app utilization

App utilization	N = 27
The app was easy to use	
Strongly Agree	12 (44%)
Agree	12 (44%)
Neutral	2 (7%)
Disagree	1 (4%)
Strongly Disagree	0 (0%)
The App was Useful	
Strongly Agree	9 (33%)
Agree	14 (52%)
Neutral	4 (15%)
Disagree	0 (0%)
Strongly Disagree	0 (0%)
How often was the app used?	
Multiple times per day	4 (15%)
Daily	3 (11%)
Weekly	11 (41%)
Monthly	7 (26%)
Never	2 (7%)

## Discussion

In this prospective, controlled, pre-post study, use of an antibiogram and treatment algorithm app was associated with higher use of hospital-specific antibiogram data and a greater improvement in knowledge scores compared to the control group. Self-reported confidence in prescribing, however, "was similar for both groups."

Multiple past studies have demonstrated that there is a strong interest in, and use of, smartphone apps among medical trainees.^11^^,^^14^^-^^21^ An increasing number of medical apps are being developed, but many are never evaluated to see what, if any, educational impact they have. Instead, many are simply studied to assess patterns of use (e.g., number of downloads, screen views).^14^^,^^20^^,^^21^ A recent single-arm case-study of an antimicrobial prescribing app found that 100% of the target population had downloaded the application within 12 months and that 71% of the participants experienced a self-reported improvement in antibiotic knowledge after accessing the application.^14^ A similar single-arm study assessed the impact of a smartphone app to aid family doctors (N=14) in treating depression.^11^ Their study found that smartphone app use improved the doctors knowledge of treating depression, but their study was limited by a small sample size (N=14) and a lack of a comparator group that did not have access to the app.^11^ Similar studies exist for apps related to other medical subspecialties (i.e., hematology, infectious diseases, plastic surgery).^8^^,^^11^^,^^13^^,^^18^

The advantages of our study were its prospective design and the inclusion of a control group. Both are crucial when trying to assess the impact of an educational program.^15^^,^^16^ Without an adequate control group and a method that accounts for the change in knowledge over time for other reasons, any observed findings might be due to unmeasured confounding factors. In our study, the inclusion of this control group was important since we expected that knowledge related to antimicrobial prescribing might increase over the course of an internal medicine rotation, even without access to an app.

One explicit limitation of our study is that clinically relevant endpoints such as appropriateness of antimicrobial therapy were not measured. This endpoint was a priori deemed to be beyond the scope of our study and would have required a substantially larger sample size to power the study adequately. Furthermore, assessing the impact of antimicrobial stewardship strategies is challenging because the definition of "appropriate therapy" is varied and controversial. One potential endpoint that has been suggested is frequency of inappropriate use of antimicrobials, but the definition of "inappropriate" can be subjective and may require consensus by expert reviewers.^1^

Our study has several other limitations. First, the knowledge assessment test was developed for the purposes of our study and has not been previously validated. Second, our study took place at one hospital which limits the generalizability of our results. Third, the duration of follow-up in our study was relatively short, and thus it is unknown whether any gains in knowledge were maintained long-term. Finally, since our study was not randomized, our results may partially be explained by unmeasured confounding factors, such as differential educational opportunities or exposure to infectious diseases related cases during the study period for the two groups.

A theoretical concern of using apps is that people might become reliant on the app and not retain important basic facts since they know they can rely on their app.^17^^,^^18^ The results of our study showed the opposite. The knowledge assessment scores were unchanged for the control group and improved in the app group. This might be because participants in the app group were exposed to the content more frequently since it was literally at their fingertips. Since medical trainees often use electronic clinical resources during their training, it is reassuring to know that these can directly improve their medical knowledge. This highlights the importance of these resources being credible and providing accurate and up to date information.

Another theoretical concern of practitioners using apps is that they might overstate or overestimate what they know since the knowledge is readily accessible.^17^^,^^18^ Our study demonstrated that the app group and the control group expressed a similar level of confidence in their knowledge of various infectious disease topics.

## Conclusions

Antimicrobial stewardship requires clinicians to be aware of both local antibiotic resistance rates and hospital-specific treatment guidelines.^2^^,^^19^ Hospitals invest a lot of time, energy, and money into developing these documents, but if they are not readily accessible, they often go unused. Our study found that the use of a smartphone app improved trainee rates of accessing antibiogram data and improved their knowledge about antibiotic prescribing. These findings reinforce the notion that smartphone apps can be a useful and innovative means of delivering medical education. In the case of this project, we created our app, but this is not necessary if there are existing high-quality apps with accurate, up to date, and relevant information. Considering the busy schedules of medical trainees, a final benefit of using apps as an adjunct to traditional forms of teaching (e.g., lectures, tutorials) is that they can be used seamlessly during clinical rotations.

### Acknowledgments

We thank Jeff Alfonsi for his help with app programming and Rosane Nisenbaum for her assistance with statistical analyses. All authors have no financial or personal relationships or affiliations that could influence the decisions and work on this manuscript. Dr. Fralick receives funding from the Eliot Phillipson Clinician-Scientist Training Program at the University of Toronto, the Clinician Investigator Program at the University of Toronto, CIHR and The Detweiller Traveling Fellowship funded by the Royal College of Physicians and Surgeons of Canada.

### Conflict of Interest

This work was supported by an unrestricted grant from Medbuy. Medbuy is a Canadian healthcare group purchasing organization. The funding organization had no role in the design and conduct of the study; collection, management, analysis, and interpretation of the data; and preparation, review, or approval of the manuscript. The app is free, does not contain advertisements, and does not provide revenue to any of the study investigators (with the exception of John Bartlett who was paid to program the app). The authors report no conflicts of interest.
